# A question of support

**DOI:** 10.7554/eLife.52881

**Published:** 2019-10-23

**Authors:** Elsa Loissel

**Affiliations:** eLifeCambridgeUnited Kingdom

**Keywords:** mental health, academia, research, survey

## Abstract

Who helps early-career researchers when they experience mental health difficulties?

Concerns about mental health in the research community have been growing in recent years. In 2014, a survey found that 47% of the PhD students at the University of California, Berkeley met the threshold for depression (The Graduate Assembly, 2014). And a study of over 3,000 graduate students in Belgium suggests that the risk for psychiatric disorders is higher in academia than in other highly-educated groups (Levecque et al., 2017). Other reports paint a similarly worrying picture (Evans et al., 2018).

These issues have brought the different types of relationships within research groups into sharp focus. When it comes to peers, early-career researchers may feel isolated and have no one at work to turn to (Barreira et al., 2018). A 2014 survey of 2,500 UK academics also highlighted a lack of support from superiors: over a third of respondents who disclosed mental health struggles to senior management did not receive any emotional or practical help (Shaw, 2014).

In response, some institutions have started to put in place measures to prevent or combat mental health problems. But on the ground, certain colleagues, members of non-academic staff and group leaders are already trying to help individuals who struggle: what are the experiences of those who provide such informal support?

## Barriers for supportive supervisors

"I think some of my major failures in the last few years have been management failures, where I look back and say: ‘I handled that completely wrong’," says Alice (names have been changed), an early-career principal investigator (PI) who has been supporting several lab members with complex mental health issues. "No one has ever taught me to be a manager. I even went on courses that I thought would teach me, but they didn’t. You have to strike the balance between getting someone to achieve their potential and not stressing them out, between being supportive and being a boss."

Managing students with mental health issues can come with additional challenges for untrained supervisors. During her talk at the I, Scientist conference in Berlin, for example, Wendy Ingram, a postdoctoral fellow at Johns Hopkins School of Public Health, told the audience that some supervisors can feel poorly equipped to help with mental health issues, and may therefore shy away from that role. PIs may also receive little help from their institutions when lab members go through a difficult time. "Unless students drop out, there are no penalties for labs where trainees are miserable," explains Alice. "But when PIs spend a lot of time supporting people or encouraging them to take leave for their issues, this may create a drop in output for a while, and that can lead to much bigger penalties for the lab."

Supervisors themselves can also struggle with their mental health, with burnout being a widespread problem (Guthrie et al., 2018). "Many early- and mid-career PIs recognize the importance of mental wellbeing for our students, in part based on our own experiences having, or having had challenges with mental wellbeing," says Roger, a US professor who got tenure a few years ago. "I think it will be challenging, if not impossible, for PIs who currently suffer to effectively support and mentor students who may potentially be suffering as well. The stigma of mental health is slowly being erased for mentees, but we are only scratching the surface or providing window dressing for faculty."

## Peers and staff: an invisible role

For early-career researchers, help may come from peers, in the form of routine, spontaneous interactions. As postdoctoral researcher Jordan recalls: "Most of the supposed support I've been giving has been letting the student next to me rant and just occasionally nodding or picking up the chocolate and sliding it over." But even this casual support can make a difference. A recent survey of PhD students in US economics departments, for instance, showed a correlation between better mental health and having good friends in the institution, although wellbeing suffered when students viewed their peers as being competitive (Barreira et al., 2018).

In fact, group members may be the only people close enough to notice early, subtle signs of a decline in wellbeing, such as insomnia, decreased appetite, irritability or restlessness. "I think it has a lot to do with empathy. Once you know someone, you can really get a feeling for who they are, and you feel when their behaviors are different," explains lab manager Jo.

I wouldn’t quite say my lab mates helped keep me alive, that’s over exaggerating. But they certainly kept me going.

Peers can therefore be the first to act when a person’s mental health takes a sharp downturn: "I wouldn't quite say my lab mates helped keep me alive, that's overexaggerating," says Jordan when discussing a severe bout of depression during their PhD. "But they certainly kept me going. These are people that you can call. Even just knowing this, it is sometimes enough to get you through."

Some of this help may take place without peers explicitly discussing it, which could be important for researchers who do not disclose their mental health conditions. "One of the first things I do, even without talking to them," explains Jo, "is to try to make more time and to help more to take some of the pressure off." Jordan also describes trying to be quietly approachable as a way to encourage people to open up.

Amongst group members, non-academic staff may also be playing a crucial role in providing support. A survey of over 700 UK technicians showed that more than half had discussed a personal problem with a postgraduate student (Technician Commitment Collaborative Team, 2019). In particular, early-career researchers may feel ‘safer’ approaching staff rather than other researchers. "I'm not another PhD student who's competing for supervisor’s time, or a paper in a high-impact journal," says Jo. "I'm outside of this whole system." Lab managers can even step up on behalf of trainees and act as a buffer during conflicts with supervisors, something that peers may not be able to do.

## Setting up informal support

Initiatives have started to emerge to formalize the support offered by peers beyond the confines of a research group. For instance, some universities are setting up mental health ambassadors programs or peer-to-peer networks, where postgraduate researchers trained in active listening and signposting are embedded within the community as first point of contact. Academic online communities such as Women in Academia Support Network, #PhDChat, PhD Balance or New PI Slack also create a supportive collegial experience regardless of lab culture and geography.

Within labs, however, it may be the role of the PIs to foster and nurture a compassionate workplace. In Jordan’s words: "It could have been a very different story if I didn't work in a multidisciplinary, very collaborative, very supportive, very friendly group. If you're taking more time to help somebody psychologically or you're leaving early because you're going to have drinks with the poor chap who has failed his experiments three times in a row, there are others who will gladly absorb your slack. This would not work in a research group where the boss is having multiple people compete on the same question." Building such groups could start, for example, with supervisors discussing mental health issues and sharing their own experiences to remove stigma, something Roger has done in his lab (see Maestre, 2019 for other suggestions).

Help from staff and colleagues may be crucial to prevent people from falling through the cracks. But this ‘on the ground’ support can only go so far: "It's everybody doing a little bit of work, and then there are professionals who are trained for the real job," says Jo. Only changes in culture as well as better mental healthcare for both early-career and senior researchers may address the root causes of academic ill-health.

At the start, this supporting role was not something that was actually given to me, or even discussed and highlighted as important.

## The needs of supporters

A recurring theme in discussions with staff and supervisors is that most of the supporting work may be shouldered by young women. "I think the general belief that men are less approachable is probably and sadly true," says Roger. This feeling is shared by Jordan and Alice, who also notices that female staff may be more likely to be appointed to supporting roles. These comments are in line with studies reporting that undergraduate students expect female professors to be more nurturing, and therefore to provide more emotional work and special treatment (El-Alayli et al., 2018; Sprague and Massoni, 2005).

People who have experienced mental health difficulties may also find themselves thrust into supporting roles. As Jordan explains: "If you have not gone through mental health issues, these experiences are not on your radar. This means that you might not notice when somebody else is going through them."

However, it remains difficult to assess the impact of support on those who provide it, because most of these interactions remain invisible. "At the start, this supporting role was not something that was actually given to me, or even discussed and highlighted as important," recalls Jo. "Nobody ever told me to give people time off when they feel burned out: I just realized it needed to happen."

Those in supporting roles may also feel differently about the demands and consequences of giving help. "It's not like 90% of the time is spent on taking care of mental health issues," says Jo. "Support happens along the way, little things, nothing big, nothing that drains me or takes away time from all the other things I have to do." But for Alice, being ‘labeled’ as a supportive individual within the institution created strains. "Being approachable is not something that comes in your workload, it's not a role that you have… It just happens," she explains, "so you can get in a situation where you spend a lot of time dealing with the needs of students and grad students, without recognition."

A 2016 report based on interviews and focus groups with 52 UK academics helping university students may give further insight into the impact of supporting roles, and what can be done to lessen it (Hughes et al., 2018). In particular, it highlights that certain situations can negatively affect the wellbeing of these supportive individuals, especially when students fail to find help within the institution. As a result, the authors provide 12 recommendations for universities, including recognizing the time spent on emotional support; creating spaces, support and training for faculty to manage the adverse impact this role can have; and clarifying their responsibilities when it comes to giving support. It remains to be seen whether peers and supervisors who help academics (rather than university students) face similar issues, and would profit from the same measures.

Ultimately, better recognition and assistance for individuals who provide support may encourage others to do the same, and could benefit the early-career researchers they help. As Jordan points out: "In the long term, a culture of support makes a research group, or an institution on any scale, a far better place to work for everyone: people do their best work when they feel valued, and respected, and are treated like humans."

## Share your experiences

Do you know someone in academia who struggles with their mental health? Have you provided support to somebody? We are interested in collecting your experiences through our survey so we can understand how to better help those who support others.

## Note

This Feature Article is part of a collection on Mental Health in Academia. Names have been changed to preserve anonymity.
